# The difference of lipid profiles between psoriasis with arthritis and psoriasis without arthritis and sex-specific downregulation of methotrexate on the apolipoprotein B/apolipoprotein A-1 ratio

**DOI:** 10.1186/s13075-021-02715-4

**Published:** 2022-01-07

**Authors:** Bing Wang, Hui Deng, Yao Hu, Ling Han, Qiong Huang, Xu Fang, Ke Yang, Siyuan Wu, Zhizhong Zheng, Nikhil Yawalkar, Zhenghua Zhang, Kexiang Yan

**Affiliations:** 1 Institute of Dermatology and Department of Dermatology, Huashan Hospital, Fudan University, No. 12 Middle Wulumuqi Road, Shanghai, 200040 China; 2 Department of Dermatology, Shanghai Jiaotong University Affiliated Sixth People’s Hospital, Shanghai, 200223 China; 3 Department of Laboratory Medicine, Huashan Hospital, Fudan University, Shanghai, 200040 China; 4 Department of Information, Huashan Hospital, Fudan University, Shanghai, 200040 China; 5 Department of Dermatology, Inselspital, Bern University Hospital, University of Bern, Bern, Switzerland

**Keywords:** Psoriatic arthritis, Psoriasis, Apolipoproteins, Lipid profiles, Methotrexate

## Abstract

**Background:**

Methotrexate (MTX) has a protective effect against cardiovascular diseases (CVD), but the mechanism is unclear.

**Objective:**

To investigate the effect of MTX on lipid profiles and the difference between psoriasis without arthritis (PsO) and psoriatic arthritis (PsA).

**Methods:**

In this prospective study, we recruited 288 psoriatic patients (136 PsA and 152 PsO) who completed 12 weeks of MTX treatment. Total cholesterol (TC), triglycerides (TG), lipoprotein A [LP(a)], high-density lipoprotein cholesterol (HDL-C), low-density lipoprotein (LDL), apolipoprotein A1 (ApoA1), and ApoB were measured.

**Results:**

Compared with sex- and age-matched healthy controls, psoriatic patients had significantly (*p* < 0.0001) higher levels of proatherogenic lipids and lower levels of anti-atherogenic lipids. PsA patients had a higher ApoB/ApoA1 ratio than PsO patients (*p* < 0.05). Stepwise regression analysis found a positive correlation between the inflammatory marker hCRP and the Psoriasis Area Severity Index (PASI), ApoB/ApoA1 ratio, BMI, and smoking. ApoB was positively associated with concomitant arthritis, diabetes, and hypertension. MTX decreased the levels of pro-atherogenic and anti-atherogenic lipids. However, a significant reduction of the ApoB/ApoA1 ratio by MTX was only observed in male patients.

**Conclusion:**

PsA patients had a significantly higher percentage of concomitant disease than PsO. The decrease of MTX on CVD might be related with sex.

**Trial registration:**

ChiCTR2000036192

## Summary

- The ApoB/ApoA1 ratio is a strong risk factor for cardiovascular disease.
- The ApoB/ApoA1 ratio was significantly higher in male and PsA patients compared to female and PsO patients, respectively.
- Methotrexate significantly decreased the ratio of ApoB to ApoA1 in male patients with psoriasis.

## Introduction

Psoriasis is a common chronic inflammatory disease characterized by keratinocyte abnormalities and immune dysfunctions. It affects about 2–3% of the world population [1]. About 5.8 to 30% psoriatic patients without arthritis (PsO) will develop psoriatic arthritis (PsA) [2, 3]. Epidemiological and clinical studies have consistently shown that psoriasis is associated with an increased cardiovascular risk [4]. The inflammatory cytokines found in psoriatic lesions may cause insulin resistance and trigger endothelial cell dysfunction leading to atherosclerosis and ultimately resulting in stroke or myocardial infarction [5]. Compelling evidence further suggests that pro-inflammatory cytokines such as interleukin (IL)-1, IL-6, and tumor necrosis factor (TNF)-α may alter the function of hepatocytes and arterial smooth muscular cells to induce alternated lipoprotein compositions, enhance expression of cellular adhesion molecules, and increase lipid deposition on arterial walls. All of these features can contribute to the development of arterial plaques [6]. Cytokines may then destabilize the plaque by promoting the rupture of fragile neo-vessels and increasing the expression of the plaque’s fibrous cap. This cascade of events may ultimately lead to plaque rupture and the formation of life-threatening thrombi [7].

Psoriasis is significantly associated with a higher prevalence and incidence of dyslipidemia [8]—a known risk factor for cardiovascular disease. Dyslipidemia is a broad term that describes any abnormality of plasma lipids including perturbations in plasma lipid levels or abnormalities in lipid composition. Multiple measurements of dyslipidemia are significantly affected and include raised triglycerides, raised LDL cholesterol, lowered HDL cholesterol, raised cholesterol, and raised lipoproteins [9–11]. Due to the remarkable heterogeneity of defining dyslipidemia, there is a lack of sex- and age-matched healthy controls. Moreover, adjustments were not made for confounding factors in most studies. Thus, the association between psoriasis and dyslipidemia still remains unclear.

Arthritis is an important determinant for psoriatic patients to develop severe vascular events in Taiwan [12]. Moreover, some arthritic patients on low-dose methotrexate (MTX) have altered blood lipids versus those not taking MTX [13]. In this prospective, cross-sectional study, we compared the differences in lipid profiles and cardiovascular risk parameters between PsA patients and sex- and age-matched healthy controls, between PsO patients and sex- and age-matched healthy controls, and between male and female psoriatic patients. Furthermore, we analyzed the effect of MTX on lipid profiles and further analyzed the influencing factors on lipid profiles and cardiovascular risk parameters after adjustments for sex, age, age at disease duration, disease duration, Psoriasis Area Severity Index (PASI), body mass area (BSA) scores at baseline, smoking, alcohol, height, weight, body mass index (BMI), hypertension, and diabetes.

## Methods

### Patients

This single-center prospective trial was performed in the Department of Dermatology, Huashan Hospital, Fudan University, between December 2, 2015, and December 2, 2019. In total, 288 psoriatic patients who received oral MTX treatment for 12 weeks and 288 sex- and age-matched healthy controls without prior medication from a medical examination center were recruited. The medical ethics committee of Huashan Hospital at Fudan University reviewed and approved the protocol (approval #MTX201501); all patients provided written informed consent. Patients aged ≥18 years were recruited from the outpatient population. The diagnosis of psoriasis and psoriatic arthritis (*n* = 136) was based on typical clinical and/or histopathological criteria and the Classification Criteria for Psoriatic Arthritis (CASPAR classification criteria), respectively. Patients who received systemic treatments (acitretin, cyclosporin, glucocorticoids) for arthritis or psoriasis at 1 month were excluded. The topical treatments had been stopped for more than 1 week before the beginning of the study. The therapeutic regimen followed the European guidelines on contraindications and restrictions on methotrexate. None of the patients used lipid-lowering drugs.

### Treatment

The initial oral MTX dose was 7.5–10 mg once weekly. The dose was increased by 2.5 mg every 2 to 4 weeks to a maximum of 15 mg weekly depending on the patient’s clinical response, side effects, and hematology/chemistry tests. If liver enzyme elevations were >2- and <3-fold, then the MTX dose was reduced by 2.5 mg weekly and administered once 2–4 weeks later. MTX treatment was stopped if the liver enzyme elevations were >3-fold [14].

### Assessments of lipid profiles and disease characteristics

Two certified dermatologists graded the severity and extent of psoriasis using the Psoriasis Area Severity Index (PASI) and body surface area (BSA) scores. Lipid profiles at baseline and 12 weeks for MTX treatment and fasting blood glucose at baseline were measured using conventional laboratory techniques at Huashan Hospital. Sex, age, age at disease onset, smoking, alcohol, hypertension, diabetes, height, weight, and body mass index (BMI) were recorded.

### Statistical analysis

Data are expressed as the means ± standard deviations (SDs). Statistical analyses were performed using the Mann-Whitney test, unpaired *t*-test, unpaired *t*-test with Welch correction, paired *t* test, *χ* [2] test, or Fisher’s exact test as appropriate. Stepwise multiple regression analysis was performed after adjustments for sex, age, age at disease onset, disease duration, height, weight, body mass index (BMI), hypertension, diabetes, smoking, alcohol consumption, and PASI/BSA scores at baseline. Data analyses were performed using Graph Pad Prism version 5 (Graph Pad Software Inc) and SPSS ver. 23.0 software (SPSS Inc., Chicago, IL, USA). A *p*-value of <.05 (or *p* < .025 after multiple test correction) was considered to be statistically significant.

## Results

### The improvement of skin lesions in psoriasis without arthritis (PsO) was superior to that of psoriasis with arthritis (PsA) by methotrexate

Table 1 summarizes the baseline and clinical characteristics according to psoriasis subtype and sex. PsA patients had a significantly older age (*p* < .0001), age at disease onset (*p* = .0052), longer disease duration (*p* = .0061), and higher percentage of hypercholesterolemia (*p* = .0087) than PsO patients. The mean PASI score at 12 W in PsO patients (*p* = .0354) was significantly lower than that in PsA patients although there was no difference in PASI score at baseline. The mean PASI (*p* = .0236) and BSA (*p* = .0105) change from baseline was significantly higher in PsO patients than that in PsA patients. In addition, the percentage of hypertension in PsA patients was significantly higher than that in PsO patients (*p* = .0029).

**Table 1:** Differences in baseline characteristics between psoriasis with and without psoriatic arthritis ^a^

Characteristic	PsA (*n*=136)	PsO (*n*=152)	*p*-value^b^	Male (*n*=189)	Female (*n*=99)	*p*-value^b^
Age, mean (SD), y	50.45 (13.12)	42.87 (15.63)	<.0001	46.79 (14.72)	45.79 (15.46)	.5627
Age at disease onset, mean (SD), y	36.00 (16.07)	31.35 (15.83)	.0052	35.15 (14.98)	30.50 (17.68)	.0021
Disease duration, mean (SD), y	14.51 (10.22)	11.52 (9.89)	.0061	11.65 (9.27)	15.39 (11.26)	.0089
Height, mean (SD), m	1.67 (0.08)	1.68 (0.08)	.8273	1.71 (0.06)	1.60 (0.06)	<.0001
Weight, mean (SD), kg	70.21 (12.28)	69.04 (13.28)	.4500	73.20 (11.43)	62.81 (12.56)	<.0001
BMI	25.01 (3.44)	24.48 (3.62)	.2158	24.85 (3.22)	24.49 (4.09)	.0865
PASI at baseline	14.04 (8.37)	13.78 (5.47)	.7491	14.74 (7.25)	12.29 (6.16)	.0045
PASI at 12W	4.58 (4.69)	3.55 (3.52)	.0354	4.41 (4.48)	3.31 (3.29)	.0288
Mean PASI change from baseline, mean (SD)	63.33 (31.50)	70.82 (28.28)	.0236	66.12 (30.27)	69.51 (29.57)	.2990
BSA at baseline	26.91 (22.64)	26.22 (17.42)	.7713	27.83 (20.08)	24.08 (19.77)	.0676
BSA at 12W	9.55 (16.80)	6.75 (12.20)	.0518	9.11 (16.28)	6.07 (10.45)	.0125
Mean BSA change from baseline, mean (SD)	60.75 (42.16)	71.38 (38.12)	.0105	65.11 (37.95)	68.74 (44.72)	.0813
Cumulative dose of MTX, mean (SD), mg	138.1 (19.85)	138.4 (20.00)	.8869	139.8 (19.36)	135.3 (20.66)	.0314
Hypertension	60 (44.1)	41 (27.0)	.0029	71 (37.6)	30 (30.3)	.2434
Diabetes	28 (20.6)	25 (16.4)	.4466	32 (16.9)	21 (21.2)	.4240
Smoking	36 (26.5)	52 (34.2)	.1613	86 (45.5)	2 (2.0)	<.0001
Alcohol consumption	36 (26.5)	44 (28.9)	.6933	74 (39.2)	6 (6.1)	<.0001
Hypercholesterolemia (>5.9 mmol/L)	23 (16.9)	10 (6.6)	.0087	14 (7.4)	19 (19.2)	.0056
Hypertriglyceridemia (>1.8 mmol/L)	51 (37.5)	42 (27.6)	.0786	61 (32.3)	32 (32.3)	1.000
Increased LDL (>3.7mmol/L)	28 (20.6)	21 (13.8)	.1574	35 (18.5)	14 (14.1)	.4106
Hyperlipoproteinemia (a) (>300mg/L)	18 (13.2)	19 (12.5)	.8620	21 (11.1)	16 (16.2)	.2662
Arthritis				86 (45.5)	50 (50.5)	0.4569
Male	86 (63.2)	103 (67.8)	.4569			

*Abbreviation*: *BMI* body mass index (calculated as weight in kilograms divided by height in meters squared), *BSA* body surface area, *PASI* Psoriasis Area Severity Index

^a^Data are presented as number (percentage) of patients unless otherwise indicated

^b^Mann-Whitney test, unpaired *t* test, or Fisher exact test was used when appropriate. *p* < .05 is considered to be statistically significant

Male patients had a significantly older age at disease onset and a lower percentage of hypertriglyceridemia (*p* = .0056) than female (*p* = .0021). Several metrics were significantly higher in male patients than female patients: mean height (*p* < .0001), weight (*p*<.0001), PASI score at baseline (*p*=.0045), PASI score at 12W (*p*=.0288), BSA score at 12W (*p*=.0125), cumulative dose of MTX (*p*=.0314), smoking (*p*<.0001), and alcohol consumption (*p*<.0001).

### Serum ApoB level and ApoB/ApoA1 ratio was significantly increased in psoriasis with arthritis compared to psoriasis without arthritis

Table 2 shows the difference in blood lipid profiles (ApoA1, ApoB, TC, LDL, HDL-C, Lp(a), TG) and cardiovascular risk parameters (ApoB/ApoA1, TC/HDL-C, LDL/HDL-C, LDL/ApoB) between PsA patients and sex- and age-matched healthy controls, between PsO patients and sex- and age-matched healthy controls, and between PsA patients and PsO patients. The mean values of serum ApoB (*p*<.0001), LDL (*p*=.0115) levels, ApoB/ApoA1 ratio (*p*<.0001), TC/HDL ratio (*p*=.0227), and LDL/HDL ratio (*p*=.0009) were significantly higher in PsA patients than those in sex- and age-matched healthy controls. However, the mean value of serum ApoA1 (*p*<.0001) was significantly lower in PsA patients than that in sex- and age-matched healthy controls. The mean values of serum ApoB (*p*<.0001), TG (*p*=.0098) levels, ApoB/ApoA1 ratio (*p*<.0001), and LDL/HDL ratio (*p*=.002) were significantly higher in PsO patients than those in sex- and age-matched healthy controls. The mean values of serum ApoA1 (*p*<.0001) level and LDL/ApoB ratio (*p*<.0001) were significantly lower in PsO patients than those in sex- and age-matched healthy controls. The mean values of serum ApoB (*p*=.0013), TC(*p*=.0013), LDL (*p*=.0229) levels, and ApoB/ApoA1 ratio (*p*=.0208) were significantly higher in PsA patients than those in PsO patients.

**Table 2:** The difference in blood lipid and cardiovascular risk parameters between psoriasis with and without psoriatic arthritis and healthy controls

	PsA (*n*=136)	HC1 (*n*=136)	PsA vs HC1 *p*-value	PsO (*n*=152)	HC2 (*n*=152)	PsO vs HC2 *p*-value	PsA vs PsO *p*-value
ApoA1, g/L	1.08 (0.18)	1.21 (0.17)	<.0001	1.07 (0.18)	1.20 (0.16)	<.0001	.4496
ApoB, g/L	0.76 (0.15)	0.65 (0.13)	<.0001	0.70 (0.17)	0.60 (0.12)	<.0001	.0013
TC, mmol/L	4.95 (0.89)	4.82 (0.82)	.1939	4.61 (0.89)	4.56 (0.71)	.6033	.0013
TG, mmol/L	1.74 (1.13)	1.45 (1.07)	.0347	1.57 (1.02)	1.29 (0.86)	.0098	.1894
HDL-C, mmol/L	1.23 (0.32)	1.26 (0.26)	.3967	1.21 (0.29)	1.27 (0.27)	.037	.4731
LDL, mmol/L	3.06 (0.77)	2.83 (0.70)	.0115	2.84 (0.81)	2.67 (0.65)	.0405	.0229
Lp(a), mg/L	147.9 (158.0)	121.5 (138.8)	.1443	139.6 (175.2)	125.0 (150.0)	.4364	.6747
ApoB:ApoA1 ratio	0.72 (0.19)	0.55 (0.13)	<.0001	0.67 (0.19)	0.51 (0.13)	<.0001	.0208
TC:HDL ratio	4.21 (1.07)	3.94 (0.92)	.0227	3.98 (1.03)	3.73 (0.92)	.0261	.0603
LDL:HDL ratio	2.62 (0.82)	2.31 (0.67)	.0009	2.47 (0.85)	2.19 (0.71)	.0022	.1309
LDL:ApoB ratio	4.03 (0.59)	4.37 (0.69)	<.0001	4.08 (0.56)	4.41 (0.55)	<.0001	.4869

*Abbreviation*: *ApoA1* apolipoprotein A1, *ApoB* apolipoprotein B, *HC* healthy controls, *HDL-C* high-density lipoprotein-cholesterol, *LDL* low-density lipoprotein, *Lp(a)* lipoprotein A, *PsA* psoriatic arthritis, *PsO* psoriasis without arthritis, *TC* total cholesterol, *TG* triglyceride

Mann-Whitney test, unpaired *t* test, and unpaired *t* test with Welch correction were used when appropriate. *p* < .025 is considered to be statistically significant

### The association of the effect of MTX on lipid profiles and cardiovascular risk parameters with psoriasis subtype and sex

Table 3 shows that MTX significantly decreased the levels of serum ApoB (*p*=.0003), TC (*p*=.0007), TG (*p*=.041), HDL-C (*p*=.037), Lp(a) (*p*=.0055), ApoB/ApoA1 ratio (*p*=.0076), and increased LDL/ApoB ratio (*p*=.025) in PsA patients. MTX significantly decreased the levels of serum ApoB (*p*<.0001), TC (*p*<.0001), HDL-C (*p*=.011), LDL (*p*=.0001), Lp(a) (*p*<.0001), and ApoB/ApoA1 (*p*=.0011). MTX increased the LDL/ApoB ratio (*p*=.0464) in PsO patients.

**Table 3:** The effect of methotrexate on lipid profiles and cardiovascular risk parameters in psoriatic patients with and without arthritis

	PsA (*n*=136, 0W)	PsA (*n*=136, 12W)	PsA (0W vs 12W) *p*-value	PsO (*n*=152,0W)	PsO (*n*=152,12W)	PsO (0W vs 12W) *p*-value
ApoA1, g/L	1.08 (0.18)	1.07 (0.17)	.1026	1.07 (0.18)	1.03 (0.15)	.0001
ApoB, g/L	0.76 (0.15)	0.72 (0.15)	.0003	0.70 (0.17)	0.64 (0.16)	<.0001
TC, mmol/L	4.95 (0.89)	4.75 (0.89)	.0007	4.61 (0.89)	4.39 (0.90)	<.0001
TG, mmol/L	1.74 (1.13)	1.61 (0.96)	.0410	1.57 (1.02)	1.63 (1.86)	.6930
HDL-C, mmol/L	1.23 (0.32)	1.20 (0.30)	.0370	1.21 (0.29)	1.17 (0.27)	.0110
LDL, mmol/L	3.06 (0.77)	2.96 (0.76)	.0632	2.84 (0.81)	2.68 (0.81)	.0001
LPA, mg/L	147.9 (158.0)	137.1 (149.5)	.0055	139.6 (175.2)	127.7 (173.9)	<.0001
ApoB:ApoA1 ratio	0.72 (0.19)	0.69 (0.17)	.0076	0.67 (0.19)	0.64 (0.18)	.0011
TC:HDL ratio	4.21 (1.07)	4.13 (0.98)	.1303	3.98 (1.03)	3.90 (0.18)	.1121
LDL:HDL ratio	2.62 (0.82)	2.59 (0.79)	.5030	2.47 (0.85)	2.40 (0.86)	.0551
LDL:ApoB ratio	4.03 (0.59)	4.11 (0.58)	.0250	4.08 (0.56)	4.14 (0.59)	.0464

*Abbreviation*: *ApoA1* apolipoprotein A1, *ApoB* apolipoprotein B, *HC* healthy controls, *HDL-C* high-density lipoprotein-cholesterol, *LDL* low-density lipoprotein, *Lp(a)* lipoprotein A, *PsA* psoriatic arthritis, *PsO* psoriasis without arthritis, *TC* total cholesterol, *TG* triglyceride

Paired *t* test, Wilcoxon-matched pairs test, Mann-Whitney test, unpaired *t* test, or unpaired *t* test with Welch correction was used when appropriate. *p* < .025 is considered to be statistically significant

Table 4 summarizes the differences and the effect of MTX on lipid profiles and cardiovascular risk parameters (ApoB/ApoA1, TC/HDL-C, LDL/HDL-C, LDL/ApoB) in male and female patients. Female patients had significantly higher levels of serum ApoA1 (*p*<.0001), TC (*p*=.0241), and HDL-C (*p*<.0001). Women had lower ApoB/ApoA1 ratio (*p*=.0004), TC/HDL ratio (*p*<.0001), and LDL/HDL ratio (*p*<.0001) than men. MTX significantly decreased the levels of serum ApoA1 (*p*=.0002), ApoB ( *p*<.0001), TC (*p*<.0001), HDL-C (*p*=.0029), LDL (*p*=.0001), Lp (a) (*p*=.0076), and ApoB/ApoA1 ratio (*p*=.0001); it increased the LDL/ApoB ratio (*p*=.0019) in men. MTX significantly decreased the levels of serum ApoB (*p*=.0214), TC (*p*=.0083), and Lp(a) (*p*=.0014) in women.

**Table 4:** The effect of methotrexate on lipid profiles and cardiovascular risk parameters in male and female psoriatic patients

	Male (*n*=189, 0W)	Male (*n*=189, 12W)	Male (0W vs 12W) *p*-value	Female (*n*=99,0W)	Female (*n*=99,12W)	Female (0W vs 12W ) *p*-value	Male vs female *p*-value
ApoA1, g/L	1.03 (0.16)	1.00 (0.13)	.0002	1.16 (0.19)	1.14 (0.17)	.1195	<.0001
ApoB, g/L	0.73 (0.17)	0.67 (0.15)	<.0001	0.72 (0.17)	0.69 (0.19)	.0214	.7756
TC, mmol/L	4.68 (0.85)	4.45 (0.83)	<.0001	4.94 (0.98)	4.76 (1.03)	.0083	.0241
TG, mmol/L	1.69 (1.06)	1.68 (1.66)	.1256	1.57 (1.10)	1.50 (1.14)	.1126	.3815
HDL-C, mmol/L	1.14 (0.26)	1.10 (0.23)	.0029	1.37 (0.32)	1.34 (0.31)	.1519	<.0001
LDL, mmol/L	2.94 (0.79)	2.79 (0.74)	.0001	2.95 (0.81)	2.87 (0.89)	.1342	.8775
Lp(a), mg/L	129.6 (148.6)	119.7 (143.9)	.0076	170.1 (195.7)	156.0 (191.8)	.0014	.0399
ApoB:ApoA1 ratio	0.72 (0.19)	0.69 (0.17)	.0001	0.64 (0.17)	0.62 (0.17)	.0762	.0004
TC:HDL-C ratio	4.26 (1.04)	4.18 (1.01)	.0512	3.76 (1.00)	3.68 (0.97)	.234	<.0001
LDL:HDL-C ratio	2.68 (0.84)	2.62 (0.82)	.1057	2.27 (0.77)	2.23 (0.79)	.4272	<.0001
LDL:ApoB ratio	4.04 (0.56)	4.13 (0.56)	.0019	4.09 (0.61)	4.13 (0.64)	.4161	.2722

*Abbreviation*: *ApoA1* apolipoprotein A1, *ApoB* apolipoprotein B, *HC* healthy controls, *HDL-C* high-density lipoprotein-cholesterol, *LDL* low-density lipoprotein, *Lp(a)* lipoprotein A, *TC* total cholesterol, *TG* triglyceride

Paired *t* test or Wilcoxon-matched pairs test was used when appropriate. *p* < .025 is considered to be statistically significant

### The association between the inflammatory marker hCRP, lipid profiles, and cardiovascular risk parameters in psoriatic patients

As shown in Table 5, stepwise regression analysis demonstrated that serum hCRP level was positively correlated with PASI score at baseline (*p*=.000), ApoB/ApoA1 ratio (*p*=.011), BMI (*p*=.003), and smoking (*p*=.015). Serum ApoA1 level was positively related with age (*p*=.006) and negatively associated with sex (*p*=.015), weight (*p*=.004), and hCRP (*p*=.006). Serum HDL level was negatively correlated with sex (*p*=.000) and weight (*p*=.014). Serum ApoB level was positively associated with diastolic blood pressure (*p*=.044), the concomitant of arthritis (*p*=.001), and diabetes (*p*=.006). Serum TC level was negatively related with sex (*p*=.031) and positively correlated with age (*p*=.000), diastolic blood pressure (*p*=.015), and the concomitant of arthritis (*p*=.002). Serum LDL level was positively associated with diastolic blood pressure (*p*=.003) and age at disease onset (*p*=.014). Serum TG level was related with diabetes (*p*=.000) and BMI (*p*=.001). ApoB/ApoA1 ratio was positively correlated with the concomitant of arthritis (*p*=.019), hCRP (*p*=.036), diastolic blood pressure (*p*=.014), and weight (*p*=.001). TC/HDL-C ratio was positively associated with sex (*p*=.006), diabetes (*p*=.008), diastolic blood pressure (*p*=.006), and BMI (*p*=.000). LDL/HDL ratio was positively related with diastolic blood pressure (*p*=.001) and weight (*p*=.000). LDL/ApoB was negatively correlated with the concomitant of hypertension (*p*=.021).

**Table 5:** The association between inflammatory marker hCRP and lipid profiles and cardiovascular risk parameters and clinic characteristics of patients with psoriasis

	Predictors	Univariate analysis			Stepwise regression analysis		
		*p*-value	*B*	95% *CI* for *B*	*p*-value	*B*	95% *CI* for *B*
hCRP	PASI score at baseline	.000	0.142	(0.084–0.200)	.000	0.144	(0.083–0.205)
	BMI	.001	0.203	(0.079–0.328)	.003	0.195	(0.065–0.324)
	ApoB/ApoA1	.000	4.009	(1.777–6.241)	.011	3.153	(0.746–5.56)
	Smoking	.022	0.821	(0.119–1.522)	.015	0.841	(0.165–1.518)
ApoA1	Sex	.000	−0.128	(−0.129 to −0.086)	.001	−0.091	(−0.142 to −0.039)
	Age	.000	0.003	(0.002–0.004)	.006	0.002	(0.001–0.004)
	Weight	.000	−0.005	(−0.006 to −0.003)	.004	−0.003	(−0.005 to −0.001)
	hCRP	.003	−0.012	(−0.02 to −0.004)	.006	−0.011	(−0.018 to −0.003)
HDL	Sex	.000	−0.228	(−0.297 to −0.159)	.000	−0.008	(−0.011 to −0.005)
	Weight	.000	−0.010	(−0.013 to −0.008)	.014	−0.096	(−0.173 to −0.019)
ApoB	Diastolic blood pressure	.003	0.003	(0.001–0.005)	.044	0.002	(0.000–0.004)
	Arthritis	.001	0.063	(0.025–0.101)	.001	0.084	(0.036–0.131)
	Diabetes	.001	0.081	(0.032–0.130)	.006	0.090	(0.026–0.154)
TC	Sex	.024	−0.252	(−0.471 to −0.033)	.031	−0.253	(0.483 to −0.023)
	Age	.000	0.017	(0.011–0.024)	.000	0.016	(0.009–0.024)
	Diastolic blood pressure	.013	0.013	(0.003–0.023)	.015	0.012	(0.002–0.021)
	Arthritis	.001	0.339	(0.133–0.545)	.002	0.225	(0.002–0.448)
LDL	Diastolic blood pressure	.001	0.015	(0.006–0.024)	.003	0.014	(0.005–0.023)
	Age at disease onset	.010	0.008	(0.002–0.013)	.014	0.008	(0.002–0.015)
TG	Diabetes	.000	0.657	(0.344–0.970)	.000	0.604	(0.025–0.095)
	BMI	.000	0.068	(0.033–0.103)	.001	0.060	(0.025–0.095)
ApoB/ApoA1	Arthritis	.021	0.052	(0.008–0.096)	.019	0.066	(0.011–0.120)
	hCRP	.000	0.015	(0.007–0.023)	.036	0.009	(0.001–0.018)
	Diastolic blood pressure	.000	0.004	(0.002–0.006)	.014	0.003	(0.001–0.005)
	Weight	.000	0.005	(0.004–0.007)	.001	0.004	(0.001–0.006)
TC/HDL-C	Sex	.000	0.504	(0.253–0.754)	.006	0.016	(0.005–0.027)
	Diabetes	.001	0.516	(0.207–0.825)	.008	0.460	(0.121–0.799)
	Diastolic blood pressure	.000	0.024	(0.013–0.036)	.006	0.016	(0.005–0.027)
	BMI	.000	0.109	(0.077–0.142)	.000	0.087	(0.050–0.125)
LDL/HDL-C	Diastolic blood pressure	.000	0.021	(0.012–0.030)	.001	0.019	(0.008–0.030)
	Weight	.000	0.025	(0.018–0.032)	.000	0.020	(0.010–0.030)
LDL/ApoB	Hypertension	.016	−0.172	(−0.310 to −0.033)	.021	-0.168	(−0.310 to −0.026)

*Abbreviation*: *ApoA1* apolipoprotein A1, *ApoB* apolipoprotein B, *HC* healthy controls, *HDL-C* high-density lipoprotein-cholesterol, *LDL* low-density lipoprotein, *Lp(a)* lipoprotein A, *TC* total cholesterol, *TG* triglyceride

Stepwise multiple regression analysis was performed after adjustment for sex, age, age at disease onset, disease duration, BMI, PASI and BSA scores at baseline, smoking, alcohol, diabetes, and hypertension. Only the significant variables are shown. *p* < .05 is considered to be statistically significant

## Discussion

Our results demonstrate that PsA and PsO patients have significantly higher levels of pro-atherogenic lipids such as ApoB and LDL and lower levels of anti-atherogenic lipids such as ApoA1 than sex- and age-matched healthy controls. The ratios of ApoB/ApoA1 and LDL/HDL-C were also significantly higher in PsA and PsO patients than in healthy controls. The ApoB/ApoA1 ratio is a strong and new risk factor for cardiovascular disease (CVD) [15]. Data from Indian patients with acute myocardial infarction (AMI) demonstrated that the ApoB/ApoA1 ratio was a better discriminator of coronary artery disease (CAD) risk than other conventional lipid ratios including the ratio of TC/HDL-C and LDL/HDL-C [16]. Moreover, the ApoB/ApoA1 ratio was associated with femoral artery atherosclerosis as measured both as intima-media thickness and plaque occurrence [17]; and carotid artery atherosclerosis measured by intima-media thickness [18]. A higher ApoB/ApoA ratio implies that more cholesterol is likely to be deposited in the arterial wall thereby provoking atherogenesis [19]. Our results are in accordance with a previous observation that psoriatic patients had a higher prevalence of ischemic heart disease (3.3% vs 1.8%, *OR* 1.87) and vascular cerebral accidents (1.8% vs 1.2%, *OR* 1.55) [20].

We report a significantly higher percentage of hypertension and hypercholesterolemia in PsA patients than PsO patients probably because the PsA cohort was older at disease onset with longer disease duration [21]. Previous studies demonstrated that psoriatic patients have a greater prevalence and incidence of hypertension. In particular, PsA and severe psoriasis were associated with a greater odds of hypertension [22]. Psoriatic patients also have increased renin-angiotensin system activity, vascular damage, and oxidative stress linked to psoriasis and hypertension [23–25].

In addition, we found that PsA patients had significantly higher levels of ApoB, TC, and LDL and a higher ApoB/ApoA1 ratio than PsO patients, which is in accordance with a previous report that PsA patients had higher burdens of specific comorbid disease than PsO patients [26]. Serum ApoB and TC levels were positively associated with arthritis after adjusting for sex, age, weight, BMI, disease duration, PASI and BSA score, diabetes, and hypertension. Our results on a higher ApoB/ApoA1 ratio in PsA differ from a recent study, which reported a higher ApoA/ApoB ratio in PsA compared to PsO [27]. Discrepancy in the study population might explain these results, since only age- but not sex-matched healthy controls were investigated in the former study. Indeed, our study found sex was also an important factor influencing lipid profiles. Male patients had significantly lower levels of TC, especially anti-atherogenic lipid profiles such as high-density lipoprotein HDL-C and ApoA1 than female patients. The ratio of ApoB/ApoA1 was significantly higher in male patients than in female patients, which was consistent with the epidemiology of cardiac and vascular disease whereby the age-adjusted CVD mortality and morbidity rates are highest in men than in women [28].

The effects of MTX on lipid profiles and CVD are controversial. One meta-analysis showed that the use of MTX predicted a reduction of 20% in the risk of cardiovascular events [29]. However, another study found that there was no effect of MTX on the levels of HDL, LDL, TG, TC, IL-1β, IL-6, and CRP. Low-dose MTX did not result in fewer cardiovascular events among patients with stable atherosclerosis than placebo. But this study did not stratify the patients by sex [30]. In our study, when patients were stratified by PsO and PsA, MTX significantly downregulated the levels of serum ApoB, TC, HDL-C, and Lp(a), as well as the ratios of ApoB/ApoA1 and LDL/ApoB in patients with PsO and PsA. Furthermore, MTX differentially reduced the levels of serum ApoA1 and LDL in patients with PsO and decreased serum TG level in patients with PsA. When patients were stratified by sex, MTX significantly downregulated the levels of serum ApoB, TC, and Lp(a) in both male and female patients with psoriasis, and differentially reduced the levels of serum ApoA1, HDL-C, LDL, and ratios of ApoB/ApoA1 and LDL/ApoB in male patients with psoriasis, but not in female patients. Therefore, the decrease of ApoB, TC, and Lp(a) by MTX was not related with sex and psoriasis subtype. However, the effect of MTX on some lipid profiles (ApoA1, HDL-C, LDL, TG) was associated with sex and psoriasis subtype. The mechanism remains to be clarified. Our another study also found MTX treatment significantly reduced the levels of serum hCRP and blood pressure in male patients, not in female patients. This indicates that the downregulation of MTX on lipid profiles was related with anti-inflammation. It has been reported that androgens can enhance the anti-inflammatory effects of MTX, which might be the main reason why the anti-inflammatory effect and downregulation of lipid profiles of MTX were more obvious in male patients than in female patients [31].

The association between inflammation and lipid profiles was further analyzed in this study. Our results demonstrated that the inflammatory biomarker hCRP was not only positively correlated with disease severity (PASI score) at baseline, but was also positively associated with ApoB/ApoA1. In addition, high BMI and smoking are known to increase serum hCRP level. This might be one reason why men had significantly lower levels of anti-atherogenic lipid profiles. The ratios of ApoB/ApoA1, TC/HDL, LDL/HDL-C, and LDL/ApoB were all regarded as cardiovascular risk parameters. But only ApoB/ApoA1 was positively correlated with inflammatory marker hCRP and the concomitant of arthritis, and they were all associated with diastolic blood pressure or the concomitant of hypertension, weight, or BMI in a stepwise regression analysis. In addition, TC/HDL-C ratio was positively related with the concomitant of diabetes. Therefore, ApoB/ApoA1 ratio is the best biomarker to predict the risk of CVD [32].

In addition, we further analyzed the association of lipid profiles with clinic characteristics of psoriasis and concomitant disease. Our data showed that ApoA1 level was negatively associated with hCRP, not HDL level. This indicates that ApoA1 is more sensitive to inflammation than HDL-C. Another study also found that serum ApoA1 level showed a strong negative correlation with some markers of systemic inflammation including serum CRP and interleukin (IL)-8 levels and blood neutrophil count [33]. ApoB is the best marker to predict concomitant disease, which was positively correlated with diastolic blood pressure, the concomitant of diabetes and arthritis. ApoB is a key component of atherogenic lipids and a more accurate measure of cardiovascular risk than LDL or non-HDL-C. ApoB was reported to aggravate arthritis by eliciting the production of TNF-α, IL-1β, and IL-6 through p38 mitogen-activated protein kinase and NF-κB pathways. ApoB is an important independent immune-modulator that links lipid metabolism with local and systemic inflammatory responses [34]. TC and LDL were positively associated with diastolic blood pressure, and TG was positively correlated with diabetes. It has been reported that TC might correlate to arterial stiffness [35] and increase the lifetime risk of coronary heart disease mortality [36]. Diabetes was reported to reduce HDL cholesterol, a predominance of small dense LDL particles, and elevated triglyceride levels [37]. Therefore, the levels of lipid profiles were related with psoriasis and its concomitant disease.

## Conclusions

In conclusion, inflammation in psoriasis can increase serum hCRP levels, resulting in the reduction of anti-atherogenic lipid ApoA1 and increase of pro-atherogenic lipid ApoB. ApoB and ApoB/ApoA1 ratio were the best biomarkers to predict inflammation and concomitant disease, especially CVD. MTX decreased pro-atherogenic lipid profiles as well as anti-atherogenic lipid profiles. The ratio of ApoB to ApoA1 was significantly downregulated in male patients with psoriasis after MTX treatment, indicating that the role of MTX on CVD and other concomitant diseases might be related to sex.

## Data Availability

Yes.

## Abbreviations

ApoA1: Apolipoprotein A1
ApoB: Apolipoprotein B
BMI: Body mass index
BSA: Body surface area
CVD: Cardiovascular disease
HC: Healthy control
HDL-C: High-density lipoprotein-cholesterol
IL: Interleukin
LDL: Low-density lipoprotein
Lp(a): Lipoprotein A
MTX: Methotrexate
PASI: Psoriasis Area Severity Index
PsA: Psoriatic arthritis
PsO: Psoriasis without arthritis
TC: Total cholesterol
TG: Triglyceride
TNF: Tumor necrosis factor
